# Triboluminescence Phenomenon Based on the Metal Complex Compounds—A Short Review

**DOI:** 10.3390/ma14237142

**Published:** 2021-11-24

**Authors:** Adam Szukalski, Adam Kabanski, Julia Goszyk, Marek Adaszynski, Milena Kaczmarska, Radoslaw Gaida, Michal Wyskiel, Jaroslaw Mysliwiec

**Affiliations:** Advanced Materials Engineering and Modelling Group, Faculty of Chemistry, Wroclaw University of Science and Technology Wyb, Wyspiańskiego 27, 50-370 Wroclaw, Poland; 246086@student.pwr.edu.pl (M.A.); 246108@student.pwr.edu.pl (M.K.); 246306@student.pwr.edu.pl (R.G.); 246214@student.pwr.edu.pl (M.W.); jaroslaw.mysliwiec@pwr.edu.pl (J.M.)

**Keywords:** triboluminescence, mechanoluminescence, copper(I) complex, manganese(II) complex, ruthenium(II) complex, europium(III) complex

## Abstract

Triboluminescence (TL) is a phenomenon of light emission resulting from the mechanical force applied to a substance. Although TL has been observed for many ages, the radiation mechanism is still under investigation. One of the exemplary compounds which possesses triboluminescent properties are copper(I) thiocyanate bipyridine triphenylphosphine complex [Cu(NCS)(py)_2_(PPh_3_)], europium tetrakis dibenzoylmethide triethylammonium EuD_4_TEA, tris(bipyridine)ruthenium(II) chloride [Ru(bpy)_3_]Cl_2_, and bis(triphenylphosphine oxide)manganese(II) bromide Mn(Ph_3_PO)_2_Br_2_. Due to the effortless synthesis route and distinct photo- and triboluminescent properties, these compounds may be useful model substances for the research on the triboluminescence mechanism. The advance of TL studies may lead to the development of a new group of sensors based on force-responsive (mechanical stimuli) materials. This review constitutes a comprehensive theoretical study containing available information about the coordination of metal complex synthesis methodologies with their physical, chemical, and spectroscopic properties.

## 1. Introduction

Triboluminescence (TL) may be obtained by rubbing, crushing, cracking, and grinding as a result of an external force interaction with the substance [1]. Thousands of TL compounds have been reported over the years. It has been estimated that due to the mechanical action, light emission can be observed in 36% of all inorganic compounds, 19% of organic compounds, 37% of aromatics, 70% of alkaloids, and probably about 50% of all crystalline substances [2]. The most well-known triboluminescent substances are sucrose [3], UO_2_(NO_3_)_2_∙H_2_O [4], quartz [1], and ZnS [5]. Since the phenomenon was described hundreds of years ago, nowadays, a significant amount of TL compounds is known. However, the universal mechanism that would allow to define physical explanation for all cases of transformation mechanical energy into light has not been discovered yet.

The development of new TL compounds is a notable step in the research of the phenomenon mechanism description. The complexes of [Cu(NCS)(py)_2_(PPh_3_)] [6], EuD_4_TEA [7], [Ru(bpy)_3_]Cl_2_ [8], and Mn(Ph_3_PO)_2_Br_2_ [9] are noteworthy exemplary substances, mainly due to their visible triboluminescence and photoluminescence [10]. All of the presented compounds have a coordination structure [6,7,8,9]. Moreover, uncomplicated preparation increases the attractiveness of described substances [6].

The implementation perspectives of TL compounds are wide. The most outstanding idea is the application of mechanical stress-responsive materials as sensing elements for the new generation of force and damage sensors [11]. The external force-inducing luminescence might also be used as an efficient energy source for the photosensitive reactions [12]. Moreover, the advancement of triboluminescence-based systems might be a chance for the development of new bioimaging techniques [13] and light-supported therapies [14].

This work constitutes a short literature review on the synthesis, spectroscopic, and physicochemical properties of the selected transition metal complexes and polymer-doped systems. Moreover, special attention has been paid to the TL measurement system and implementation perspectives of the triboluminescent compounds.

## 2. The (Un)known Mechanism of Triboluminescence

The triboluminescence seems to be an uncomplicated phenomenon. Unfortunately, precise observation of TL is difficult due to many variables and its short duration. Because of the diversified properties of TL compounds and acquired divergent results, the universal triboluminescence mechanism has not been described yet. The crystals undergo elastic and plastic deformations and then crumble during cracking, which creates new surfaces exposed to external stimuli. The electric potential can be generated by charged fracture planes during the movement of charged particles or even by the potential difference between the crystal and crushing tool [15]. Impurities also affect the crystal symmetry and charge distribution. Furthermore, in order to study triboluminescence, it is necessary to take into account the environment in which the crystal is located, in particular the pressure and type of surrounding gas [13]. The relatively small progress in the understanding of triboluminescence is not too surprising, given so many variables. Many factors influencing the described phenomenon have been investigated, and several mechanisms of triboluminescence have been proposed, based on the obtained results.

Initially, the link between triboluminescence and piezoelectric crystals was sought [15]. Mechanical force creates a potential difference, which causes the electron movement. As a result, the surrounding gas is excited [1]. This theory was confirmed for the first sucrose triboluminescence, which was recorded in 1922 [13]. It was noticed that it is identical to the emission spectra of N_2_ gas discharge. The experiment was repeated under controlled conditions, in which the surrounding gas was neon. The sucrose was crushed again and, as a result, the emission of red light was observed [14]. It was also noted that the triboluminescence spectrum of photoluminescent substances consists of two parts: (i) identical to the photoluminescent spectrum and (ii) identical to the other spectrum of the excited gas [16,17]. Based on that, the following mechanism was proposed. If the triboluminescent substance also characterizes the photoluminescent properties, the excited gas will induce the PL of the substance. However, many cases contradict the described theory. Indeed, there are compounds whose triboluminescent spectrum is identical to that of the photoluminescent one, but without any hint of gas excitation [18]. On the other hand, there are many piezoelectric materials in nature that do not exhibit triboluminescence at all [1].

All tested solids create new surfaces and release electrons during fracture. The most photoluminescent materials can be excited for less than 5 eV, in comparison to nitrogen, where 11 eV are needed to excite. On this basis, it was concluded that the electrons released during the fracture cause photoluminescence and also gas excitation, if possible [1]. If the compound does not show photoluminescence, only the surrounding gas is excited. There are many tribo-photoluminescent compounds with optimal excitation values below 300 nm. Therefore, it seems impossible for the compound excitation to occur because of gas discharge, since the area of gas discharge emission is extremely weak in this range, which confirms the described theory [1]. The proposed mechanism works for most triboluminescent compounds, but there are some exceptions. For example, quartz exhibits visible orange triboluminescence in the air [1].

The explanation of TL, which would be comprehensive for all types of compounds, still has not been described. The advances in the triboluminescence phenomenon understanding will enable to design of the materials and systems exhibiting specific TL properties, which undoubtedly will pave the way for new implementation possibilities.

## 3. Synthesis of Triboluminescent Complex Compounds

One of the most significant advantages of this group of complex compounds is its relatively fast (several hours) and uncomplicated preparation. The accessible and cheap substrates and direct synthesis methods create new possibilities of process optimization. Further research may affect the future implementation of triboluminescent substances in various materials and areas.

### 3.1. [Cu(NCS)(py)_2_(PPh_3_)] Complex

There are two main preparation methods of the [Cu(NCS)(py)_2_(PPh_3_)] complex currently described in the literature, i.e., one- and two-step processes [6,16]. Despite the superficial differences, the presented chemical reactions are based on the same general type of formation.

**Figure 1:**
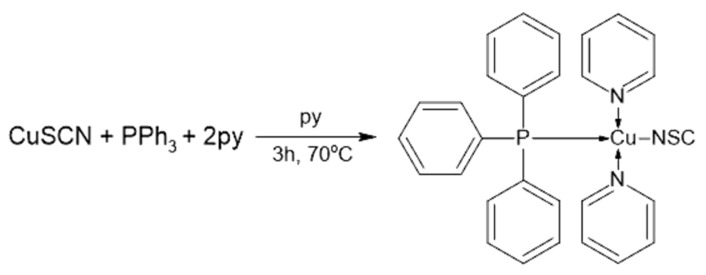
One-step synthesis reaction of the [Cu(NCS)(py)_2_(PPh_3_)] complex. Reprinted (adapted) with permission from (J. Chem. Educ. 2012, 89, 5, 652–655). Copyright (2012) American Chemical Society [6].

The first discussed synthesis method is a one-step process schematically presented in Figure 1. According to the provided literature information, 5 mL of hot pyridine containing dissolved CuSCN (0.121 g; 1.0 mmol) and PPh_3_ (0.262 g; 1.0 mmol) were mixed for 3 h in 70 °C. It is important to maintain the temperature; otherwise, the [Cu(NCS)(py)_2_(PPh_3_)] complex will not form. Carrying out the synthesis at room temperature leads to the precipitation of the [Cu(NCS)(PPh_3_)] complex [6]. Afterward, the solution was slowly cooled down, and half of the solvent was evaporated under the fume hood. During the evaporation process, the pale yellow crystals started to precipitate, and they were subsequently filtered out [6]. It is optional to cleanse the product with toluene [16].

The reaction yield can reach even 80–90%, and it is possible to obtain well-formed, colorless crystals by slowly cooling down the mixture from previously implemented higher temperature (80 °C) [1]. The one-step synthesis method is the most frequently repeated one [6,10,17].

### 3.2. EuD_4_TEA Complex

**Figure 2:**
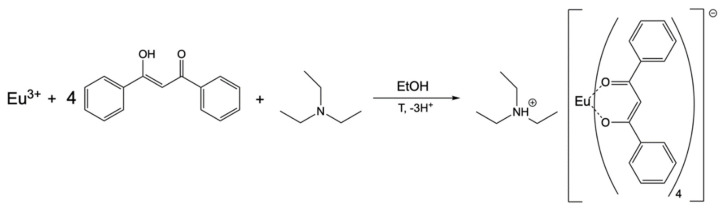
Synthesis reaction of the EuD_4_TEA complex according to [20].

Hurt et al. were the first who synthesized the europium complex in 1966. They used anhydrous europium chloride as a source of europium(III) ions [7]. In 2011, the europium chloride was replaced with europium(III) nitrate to avoid chloride contamination in the solution [19]. This modification significantly reduced the cost of the synthesis and contributed to the improvement of TL properties of the europium(III) complex [19]. The discussed synthesis is presented in Figure 2 [20]. According to Fontenot R.S. et al. [20], to obtain the EuD_4_TEA complex, firstly, europium(III) nitrate (1.44 g, 4.0 mmol) was dissolved in 25 mL of heated anhydrous ethyl alcohol. Afterward, dibenzoylmethane (2.93 g, 13.0 mmol) and triethylamine (2 mL, 14.0 mmol) were added to the solution and heated up to obtain a clear solution. The mixture was left at room temperature to cool down. During a slow temperature decrease, TL crystals were formed. The following day, the product was filtered out and left to dry.

Furthermore, it is possible to modify the synthesis process of the TL europium complex by using different solvents [21]. Dissolving europium(III) nitrate in acetonitrile or acetone speeds up the synthesis, so it is not necessary to heat the solution. Using acetone leads to the precipitation of a small crystalline product, but after harvesting it and leaving it to crystalize, well-formed crystals are obtained. The solvent has an impact on the intensity of triboluminescence and decay time of EuD_4_TEA [21]. Additionally, it was found that replacing anhydrous ethanol with laboratory ethanol has no impact on the TL results [21].

Moreover, doping EuD_4_TEA complex with organic substances such as piperine, DMMP, TEPS [20], uranium [22], or even multivitamin [23] affect TL properties, which is a useful feature for further application as an element of sensors. The influence of organic dopants will be discussed in chapter 6.

### 3.3. [Ru(bpy)_3_]Cl_2_ Complex

**Figure 3:**
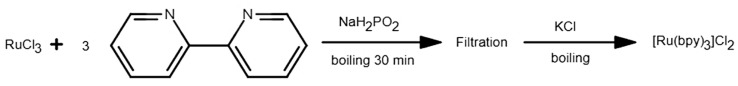
Synthesis of the [Ru(bpy)_3_]Cl_2_ complex [8].

In some publications, authors used the purchased [Ru(bpy)_3_]Cl_2_ complex [24,25], although it can be synthesized in several different ways. Sumana Bhar and Rajakumar Ananthakrishnan prepared the Ru(II)-metal complex with 70% yield by following the method in [8]. They dissolved 2,2′-bipyridine (0.234 g, 1.5 mmol) and anhydrous RuCl_3_ (0.104 g, 0.5 mmol) in distilled water. Then, freshly prepared sodium phosphinate solution was added dropwise. The mixture was boiled for 30 min under reflux. During the reaction, the color of the solution gradually changed from bluish-green to orange-red. The solution with the crude product was filtrated and KCl (3.728 g, 0.05 mol) was added to the filtrate. The mixture was heated up to the boiling point and stirred for a few minutes to obtain a deep red solution. After cooling down to room temperature red product crystallized out of the solution. The crystals were filtered, then washed from the residual impurities with ice-cold 10% aqueous acetone and acetone. In the end, the crystals were air-dried. The whole process is schematically presented in Figure 3.

In the literature, similar synthesis routes can be found, in which some parameters, such as reaction time, reducing agent, solvent, and inert gas, have been changed [25,26,27]. Frequently, the complex is given only as an intermediate product for which the yield is not given [2].

### 3.4. Mn(Ph_3_PO)_2_Br_2_ Complex

**Figure 4:**
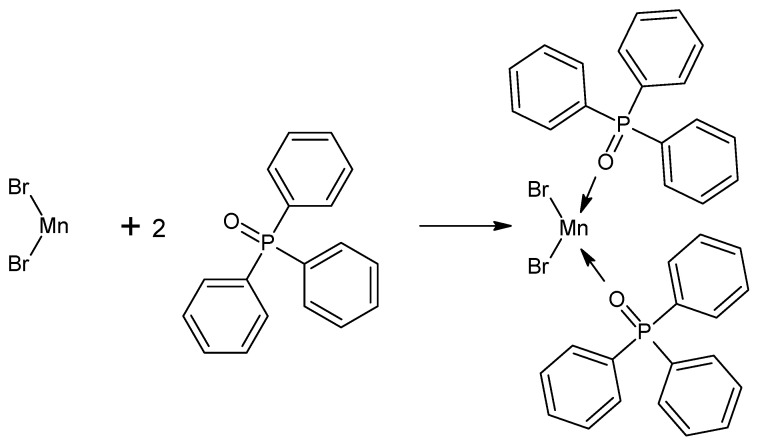
Formation of the manganese(II) complex according to [9].

This compound was synthetized in all known publications based on a method reported by Goodgame D.L. et al. [9]. The manganese complex with bromides and triphenylphosphine was prepared by the one-pot method shown in Figure 4. The phosphine oxide and manganous salt are mixed in a 2.2:1 molar ratio and then dissolved in 20–40 mL hot absolute ethanol. The mixture was left to cool down and precipitate crystals. The product was filtered out, washed with cold ethanol, and vacuum dried. The achieved yield was equal to 60% [9].

Infrared spectroscopy (IR) analysis of the product showed signals of P-O stretching—1163 cm^−1^ and 1158 cm^−1^, which confirms the presence of the phosphine oxide in the manganese(II) complex [9]. In the literature, another method was also described [25]. In fact, this method is an extension of the method described by Goodgame D.L. et al. [9]. First, 0.13 mol of triphenylphosphine oxide and 0.04 mol of MBr_2_ tetrahydrate were dissolved in hot ethanol. Reagents were exactly stirred and refluxed at 60 °C for 1 h. Next, the product was slowly cooled down to room temperature. The final mixture was filtered and left in the ambient conditions for few days. After that, precipitated light green crystals were filtered out, washed with cold anhydrous alcohol, and left to dry [25].

It is also possible to add some dopants to the crystals, for example HBr and HgCl_2_ [25]. In this case, in the first step, solutions of admixtures are added to the reaction mixture. The rest of the procedure is the same as for the synthesis of pure Mn(Ph_3_PO)Br_2_ [25].

## 4. Chemical and Structural Analysis

Analysis of the fundamental structural properties may give an opportunity to describe the details of the triboluminescence mechanism. Moreover, sufficient chemical and physical properties are necessary for the application of given compounds.

### 4.1. [Cu(NCS)(py)_2_(PPh_3_)] Complex

**Figure 5:**
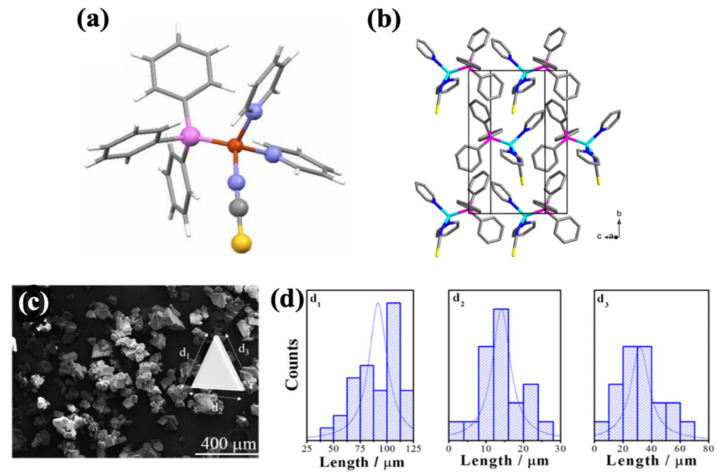
(**a**) Chemical structure of the copper complex molecule [6]; (**b**) molecular orientation of the copper complex in the crystal cell [2]; (**c**) SEM image of the crystal habit of the complex [28]; (**d**) particles’ size distribution [28]. Reprinted (adapted) with permission from (J. Chem. Educ. 2012, 89, 5, 652–655 and J. Phys. Chem. C 2017, 121, 21, 11709–11716). Copyright (2012) American Chemical Society [6,28].

The crystallographic structure of a triboluminescent compound may have a crucial influence on the TL properties [18]. In the case of the described compound, the Cu^+^ ion is surrounded by four electron-donor ligands: thiocyanate group, triphenylphosphine, and two pyridine molecules (Figure 5a,b). The coordination number is equal to 4. The NCS^−^ group is bonded with the central atom by one electron pair of nitrogen atom [28].

The morphology was described according to the scanning electron microscopy (SEM) technique. The crystals have a triangular shape, of which the sides are characterized by Gaussian distribution (Figure 5c). Side lengths were estimated in the range of (Figure 5d):

- d_1_: 100–25 µm;
- d_2_: 15–10 µm;
- d_3_: 40–20 µm [28].

The center of the molecule with Cu^+^ ion has distorted tetrahedral geometry [28]. The summarized values of the bond lengths and angles are presented in Table 1.

Copper complex crystallizes in the *P21* space group, which is part of the monoclinic crystal system [28]. This structure is characterized by a lack of symmetry centers, which is essential to enable the triboluminescence phenomenon. The edge lengths of an oblique rectangular prism are as follows:

- a = 9.4006(4) Å;
- b = 15.1492(7) Å;
- c = 10.2153(4) Å.

The angle of a slope between the sides and base is equal to β = 116.9660(10)° [28].

**Figure 6:**
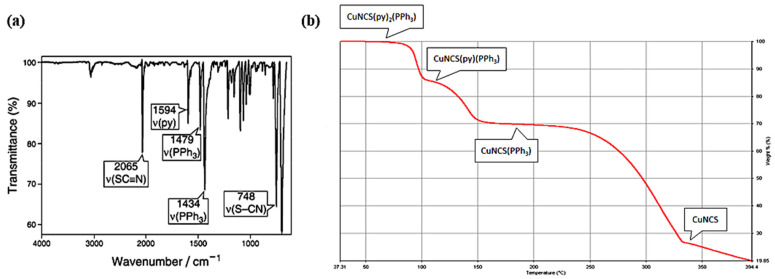
(**a**) IR spectrum of the [Cu(NCS)(py)_2_(PPh_3_)] complex; (**b**) thermogram of the [Cu(NCS)(py)_2_(PPh_3_)] decomposition process. Reprinted (adapted) with permission from (J. Chem. Educ. 2012, 89, 5, 652–655). Copyright (2012) American Chemical Society [6].

Infrared spectroscopy (IR) was used to perform the structural analysis (Figure 6a). The substance exhibits strong absorption of pyridine at 1594 cm^−1^ and two absorption peaks at 2065 and 748 cm^−1^, which is characteristic of NCS ligand, where the moiety is linked to the copper via the Cu-N bond [6]. The signals localized at 1479 and 1434 cm^−1^ are characteristic of triphenylphosphine [6]. The solid-state NMR spectroscopy method was also used. ^31^P CPMAS (cross-polarization magic-angle spinning) was also performed and confirmed the structure of the compound [10]. The obtained ^31^P CPMAS parameters are as follows: ρ = −7.2; Δν_1_ = 1537; Δν_2_ = 1568; Δν_3_ = 1581; <Δν> = 1526; d = −11.00 Hz; dν_Cu_ = 0.79 × 10^−9^.

The described copper complex exhibits sufficient thermal stability (Figure 6b) up to the temperature of 75 °C. Above this value, the sample weight reduction is observed, which is related to the complex decomposition. The first step of the degradation process is based on one pyridine ligand detachment. Next, at a temperature range of 114–120 °C, the second pyridine moiety is being detached. The final step of the copper complex thermal degradation occurs in a range of 150–330 °C, where the PPh_3_ and CuNCS parts, are being separated. At the higher temperature, the colorless CuNCS is stable [6]. According to the literature [16], the melting point of the considered compound was observed at 165–167 °C.

The solubility of the copper complex was investigated as well. Its crystals dissolve in pyridine, chlorinated solvents, acetonitrile, DMSO [6], and chloroform [16]. However, the compound is resistant to moisture and air [6].

### 4.2. EuD_4_TEA Complex

**Figure 7:**
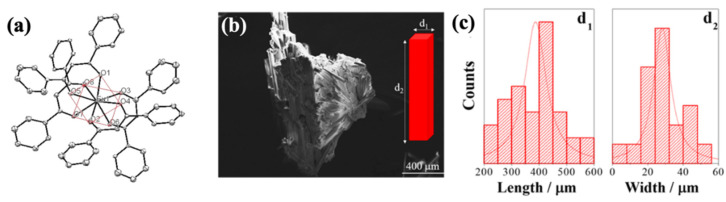
(**a**) Chemical structure of the EuD_4_TEA complex molecule; (**b**) SEM image of EuD_4_TEA crystal habit of the complex; (**c**) particles’ size distribution of EuD_4_TEA complex (d_1_—length, d_2_—width). Reprinted (adapted) with permission from (ACS Appl. Mater. Interfaces 2017, 9, 7, 6488–6496). Copyright (2012) American Chemical Society [29].

The Eu^3+^ ion is coordinated with eight dibenzoylmethane’s oxygen atoms, so the coordination number of EuD_4_TEA is equal to 8 [29]. Those coordination bonds create tetrabidentate anionic europium(III) complex. To equalize the charge of the molecule the structure is protonated with triethylammonium molecule (Et_3_NH^+^) [29]. The protonated tertiary ammonium group is responsible for its strong triboluminescent properties [7]. The molecule structure is presented in Figure 7a [29]. The hydrogen bonds between the Et_3_NH^+^ group and oxygen have a strong impact on stabilizing the long-range structure of the complex. The EuD_4_TEA was investigated using scanning electron microscopy (SEM) [29]. The SEM imagine (Figure 7b) represents a large agglomerate, which is built with smaller rectangular crystals. The size is characterized by Gaussian distribution (Figure 7c). According to the data, the average size estimates are 400–30 µm [29].

The structure of the EuD_4_TEA complex was solved and the space group was defined as *P21*, which is part of the monoclinic crystal system. The coordination geometry is square-antiprismatic [29]. Details of molecule geometry are presented in Table 2.

The unit cell dimensions are as follows:

- a = 9.0297(7) Å;
- b = 24.830(3) Å;
- c = 25.203(2) Å [29].

The angle of a slope between sides and the base is equal to β = 91.323(3)°, and the volume is 5649.2(9)° Å^3^ [29].

**Figure 8:**
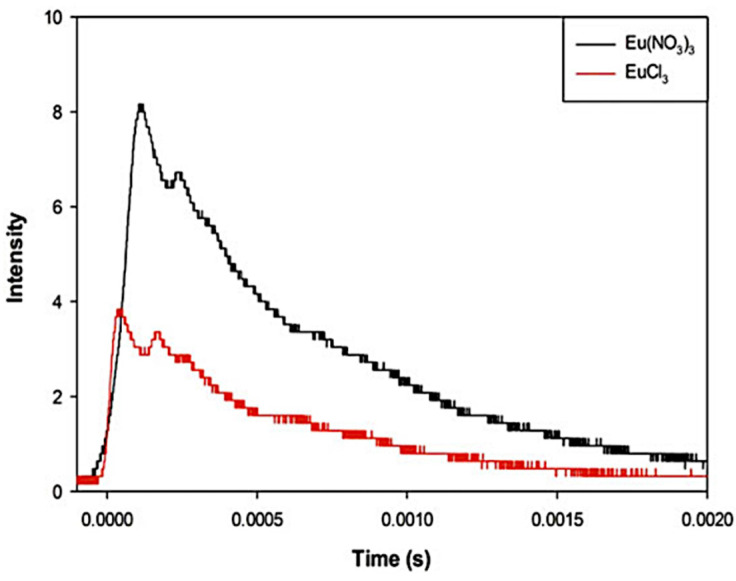
TL of EuD_4_TEA complex precipitated in synthesis with Eu(NO_3_)_3_ (black line) or with EuCl_3_ (red line) [30].

The TL properties of the described EuD_4_TEA complex depend on the europium salt used in the synthesis [19]. The use of the europium(III) chloride not only extends the synthesis time and increases the cost, but also deteriorates TL emission. Two of the same parallel syntheses, which are differing only in the use of europium(III) salt, were made. In the reaction ethanol was used as a solvent. TL spectra of the products were measured (Figure 8) [19]. The intensity of emission increased by 82% for EuD_4_TEA crystals doped with europium(III) nitrate. Moreover, the yield of synthesis increased, because the unnecessary step of washing out chlorides was omitted [19].

### 4.3. [Ru(bpy)_3_]Cl_2_ Complex

**Figure 9:**
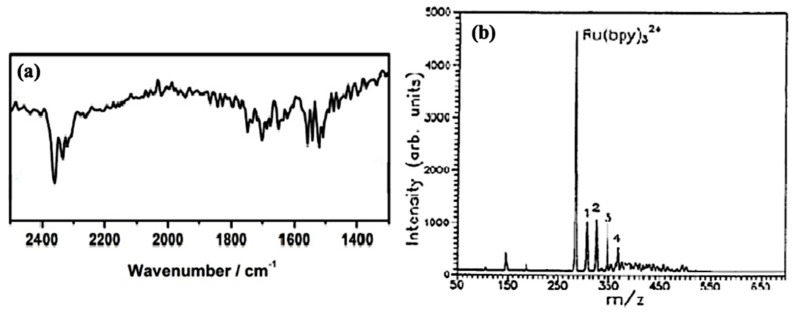
(**a**) IR spectra of [Ru(bpy)_3_]Cl_2_ [31]. (**b**) Electrospray ionization mass spectroscopy of Ru(bpy)_3_^2+^ with low level of collisional activation. Reprinted (adapted) with permission from (*J. Am. Chem. SOC.* 1990, 112, 5348–5349). Copyright (2012) American Chemical Society [32].

**Figure 10:**
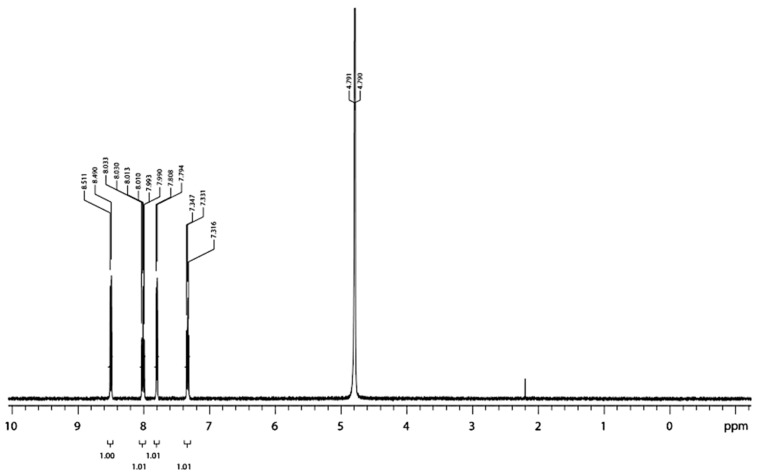
^1^H NMR spectra (400 MHz) of [Ru(bpy)_3_]^2+^ in D_2_O at room temperature. Referenced to HDO peak at 4.79 ppm. Reprinted (adapted) with permission from (*Inorg. Chem.* 2020, *59*, 3942−3953). Copyright (2012) American Chemical Society [24].

Ru(II)-metal complex is a valuable material for triboluminescence investigation, especially due to the well-described structural properties [24,31,32]. The substance shows a strong absorption band around 2370 cm^−1^, and several minor bands between 1500 cm^−1^ and 1800 cm^−1^ (Figure 9a) [31]. Transition metal complexes are difficult to analyze by mass spectrometry mainly due to their tendency to reduction during ionization and low volatility. Viswanatham Katta et al. analyzed the ions of transition metal complexes with electrospray ionization. They obtained spectra of [Ru(bpy)_3_]Cl_2_ (M = 641 g/mol) by electrospraying a 15 pmol/pL solution in acetonitrile. Figure 9b shows MS spectra with a low level of collisional activation; the peak at *m*/*z* 285 corresponds to the Ru(bpy)_3_^2+^ ion [32]. Full ^1^H NMR spectrum (400 MHz) of Ru(II)-metal complex in D_2_O was obtained at room temperature. The clear signals coming from the 2,2′-bipyridine system were found (Figure 10) [24].

**Figure 11:**
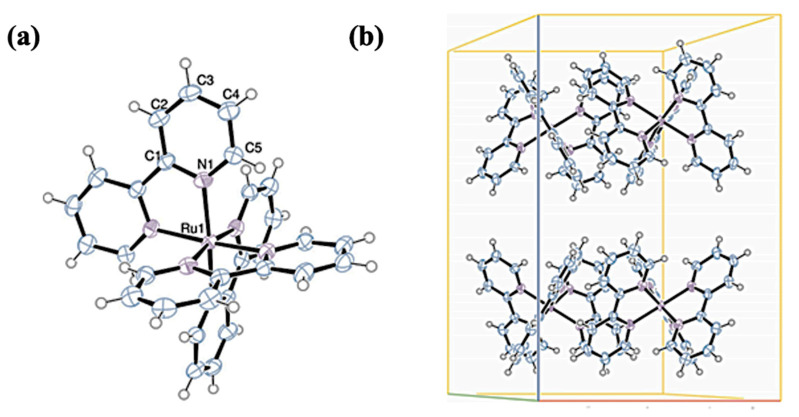
(**a**) Chemical structure and (**b**) crystal-packing diagram (H_2_O molecules were omitted in this diagram). Reproduced with permission of the International Union of Crystallography [33].

Kian Sing Low et al. analyzed a few Ru and Fe complexes by X-ray diffractometer. In [Ru(bpy)_3_]Cl_2_, the ruthenium ion is surrounded by three bidentate bipyridyl ligands, which are electron donors. The coordination number of the central atom is equal to 6. The chemical structure and crystal-packing diagram are presented in Figure 11.

The ruthenium complex exhibits typical octahedral deformation, the bond lengths are Ru–N [2.065 Å], C–C [1.454 Å], N–C [1.345 and 1.366 Å], N–Ru–N bite angle is 79.0°, and N–C–C–N torsion angle is [−4.5°] and [−5.0°]. Other data are collected in the Table 3.

### 4.4. Mn(Ph_3_PO)Br_2_ Complex

**Figure 12:**
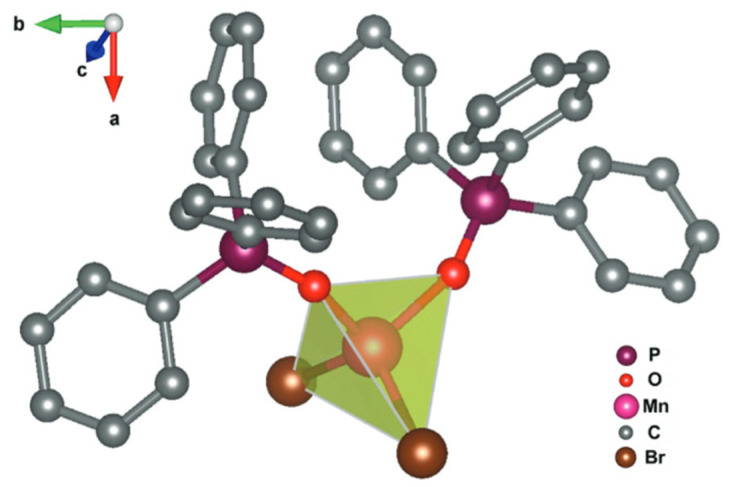
Chemical structure of the manganese complex molecule [34].

The described complex contains the Mn^+II^ ion surrounded by two Br^−^ ions and two triphenylphosphine oxide, which are bonded with the metal atom by oxygen atoms (Figure 12). The coordination number is equal to 4. Molecules of the Mn(Ph_3_PO)_2_Br_2_ crystallize as a mononuclear structure in a polar space group *P1*, so the manganese(II) complex has a non-centrosymmetric structure. Cell parameters were defined as follows:

- a = 10.013(7) Å;
- b = 10.253(7) Å;
- c = 10.564(6) Å.

Additional data are collected in the Table 4. Unit cell angles are as follows: α = 65.31(6)°, β = 63.75(5)° γ = 89.72(7)° [34].

The FTIR analysis of the products showed a peak at 1155 cm^−1^, which is caused by stretching of the P=O group. Two bands at 3051 and 1595 cm^−1^ indicate the presence of aromatic rings in triphenylphosphine. Another doublet signal at 726 and 686 cm^−1^ is caused by Mn–O stretching and bending [25].

## 5. Photo- and Triboluminescence Phenomena

### 5.1. TL and PL of the [Cu(NCS)(py)_2_(PPh_3_)] Complex

**Figure 13:**
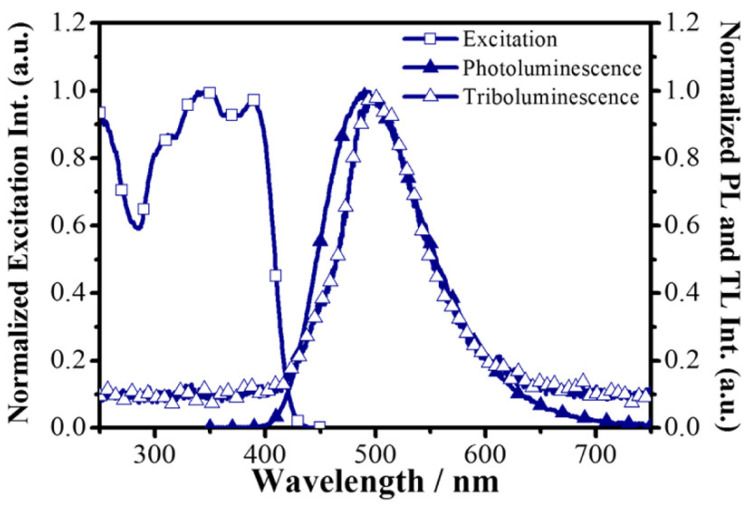
PL (fulfill triangles) and TL (empty triangles) spectra of the [Cu(NCS)(py)_2_(PPh_3_)] complex; the acquired excitation spectrum is shown as empty squares. Reprinted (adapted) with permission from (J. Phys. Chem. C 2017, 121, 21, 11709–11716). Copyright (2017) American Chemical Society [28].

The photoluminescent properties of the [Cu(NCS)(py)_2_(PPh_3_)] were first visually assessed. It is possible to observe strong photoluminescence of crystals with an excitation wavelength of 365 nm. Claudio Pettinari et al. compared the photoluminescence properties of the several copper complexes, including [Cu(NCS)(py)_2_(PPh_3_)] [10]. Each of them showed a broad absorption band localized at 370–490 nm. The maximum of the emission band was observed at 496 nm (Figure 13) [28]. Additionally, the authors measured the emission decay time, which was 5.8 ns [10].

In another work, two emission bands were observed—at 479 nm and 587 nm [16]. All of the photoluminescence spectra presented in the literature coincide to a large extent; however, minor differences occurred. It may be due to the distinctions in the purity of the tested compounds, measurement conditions, and used apparatus.

**Figure 14:**
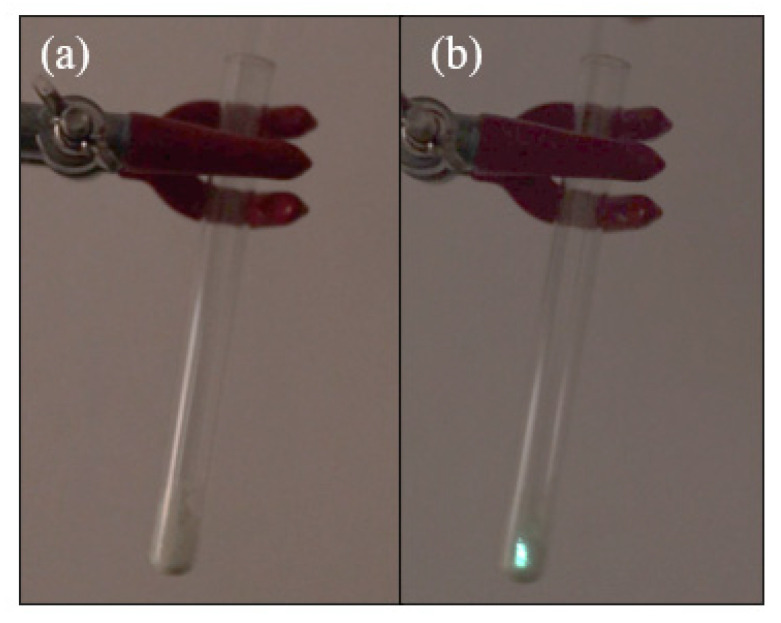
Triboluminescence of the [Cu(NCS)(py)_2_(PPh_3_)] complex: (**a**) sample of the complex without an external force applied; (**b**) visible triboluminescence obtained by crushing crystals with a glass rod.

The [Cu(NCS)(py)_2_(PPh_3_)] complex shows one of the strongest triboluminescent properties among the already known compounds, which makes their observation possible in daylight. The emitted light takes a blue-green color (Figure 14) [6].

The triboluminescence spectrum is shifted by 10 nm towards lower energy compared to the photoluminescence one; thus, maximum emission of TL occurs at 500 nm (Figure 13). The full width at half-maximum (FWHM) of the triboluminescence spectrum is 90 nm, which is 23 nm smaller than the same parameter of the PL. No other significant differences were noted between the intensity of the triboluminescence and photoluminescence emission [28]. Measurements were performed using a specially designed system described in Section 7 of the article.

### 5.2. TL and PL of the Europium Complex

Under UV light irradiation, strong red photoluminescence of the EuD_4_TEA complex is observed. The wavelength of the maximum emission is 612 nm, which corresponds to ^5^D_0_ → ^7^F_2_ transition in Eu^3+^ ions. Additionally, transitions from ^5^D_0_ to ^7^F_1_, ^7^F_3_, and ^7^F_4_ occur [23].

**Figure 15:**
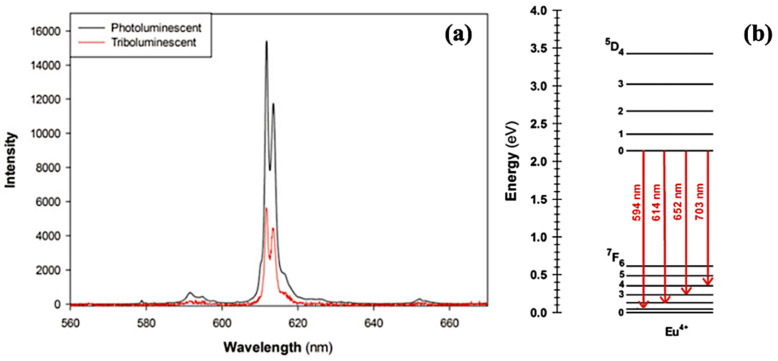
(**a**) TL (red line) and PL (black line) emission spectra of EuD_4_TEA complex. (**b**) Energy diagram of Eu^4+^ [35].

The light emitted during the mechanical deformation of crystals takes a red color, and it is caused by the typical Eu^4+^-centered ^5^D_0_ → ^7^F^0−4^ transitions, but mainly ^5^D_0_ → ^7^F_2_ transitions (Figure 15b) [35]. The TL and PL spectra are presented in Figure 15a [35]. Maximum emission of TL occurs at 614 nm. The obtained spectra exhibit significant similarities; however, TL has a lower intensity. Due to the end of the spectrometer’s measuring range, the signal coming from ^5^D_0_ → ^7^F_4_ transition has not been recorded [35].

**Figure 16:**
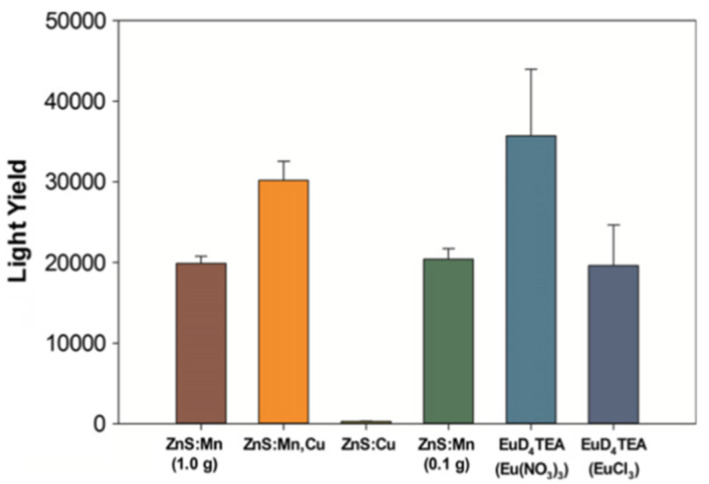
TL light yield results for chosen materials. Reprinted from: Fontenot, R.S.; Hollerman, W.A.; Aggarwal, M.D.; Bhat, K.N.; Goedeke, S.M.A. versatile low-cost laboratory apparatus for testing triboluminescent materials. Measurement. 2012, 45, 431–436. Reprinted with permission from Elsevier [36].

The EuD_4_TEA exhibits one of the highest triboluminescence intensities among all currently known substances. The bright emission is noticeable in daylight and the intensity is 106% stronger than the most popular triboluminescent compound—ZnS:Mn [36]. The relative light yield of the europium(III) complex is 42 146 ± 7784 and ZnS:Mn 20 426 ± 1294 (Figure 16). The described measurements were performed with samples made with europium(III) nitrate [36].

### 5.3. TL and PL of the [Ru(bpy)_3_]Cl_2_ Complex

**Figure 17:**
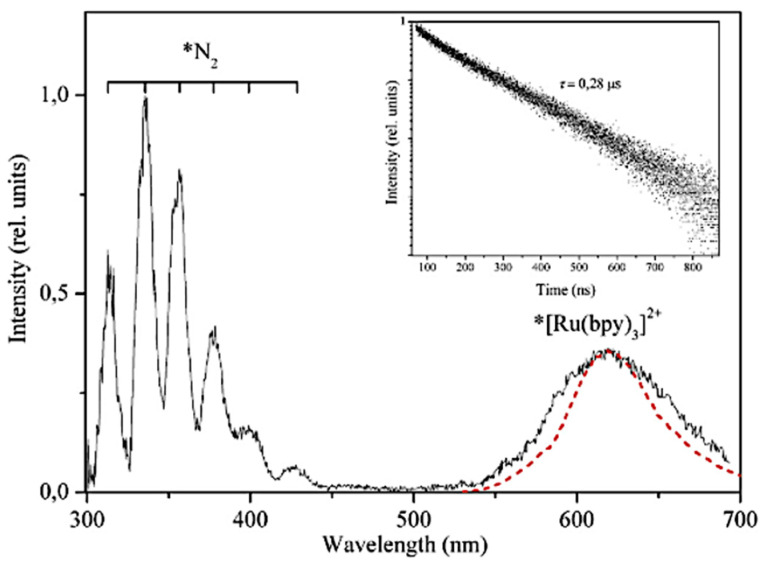
(Solid line) TL and (dash line) PL spectra of the ground [Ru(bpy)_3_]Cl_2_. Reprinted from: Sharipov G.L.; Tukhbatullin A.A.; Triboluminescence of tris(2,2′-bipyridyl)ruthenium(II) dichloride hexahydrate. Jour. of Lum. 2019, 215, 116691. Reprinted with permission from Elsevier [38].

Photoluminescent properties are observed in the [Ru(bpy)_3_]Cl_2_⋯6H_2_O complex because of the presence of the [Ru(bpy)_3_]^2+^ cations [24]. Glyus L. Sharipov and Adis A. Tukhbatullin described fundamental spectroscopic properties of the obtained complex. The measured maximum of the emission upon 450 nm excitation wavelength was 519 nm [37]. The lifetime of the excited ionic state marked as *N_2_, *[R(bpy)]^2+^ was 40 μs (Figure 17). Kalyanasundaram K. reported the quantum yield of the described complex in an aqueous solution as 0.044 [38].

The triboluminescence spectrum of [Ru(bpy)_3_]Cl_2_ ∙ 6H_2_O consists of two parts (Figure 17). The first one comes from the emission of N_2_, while the second one is similar to the photoluminescence of the complex. The similarity of the TL and PL spectra is caused by the *[Ru(bpy)_3_]^2+^ ion excitation; however, the excitation mechanisms are different [37]. The theories of the triboluminescence fundamentals are described in Section 2.

[Ru(bpy)_3_]Cl_2_⋯6H_2_O crystals are asymmetric [38], which makes this compound a particularly valuable material for TL investigation. According to one of the postulated theories, only centrosymmetric compounds exhibit triboluminescence, so the non-centrosymmetric crystal with strong triboluminescence sheds new light on the topic [38]. Moreover, the correlation between the degree of fragmentation and TL intensity has not been observed [37], which has been reported for centrosymmetric crystals [39].

**Figure 18:**
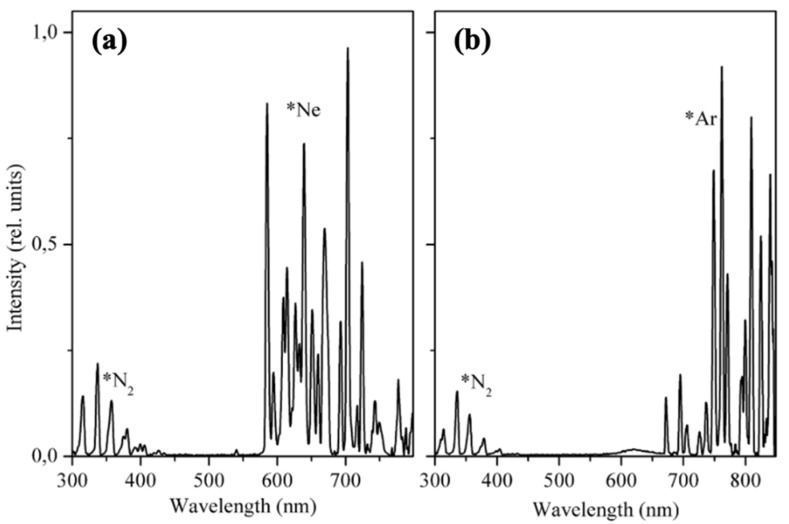
TL spectra of [Ru(bpy)_3_]Cl_2_ in: (**a**) neon and (**b**) argon atmosphere. The emission peaks corresponding to the excited gases are marked with *N_2_, *Ne, *Ar symbols. Reprinted with permission from Elsevier [37].

Glyus L. Sharipov and Adis A. Tukhbatullin reported triboluminescence spectra in specific gas environments, such as neon and argon (Figure 18). The obtained TL spectrum under neon exhibits intense emission corresponding to excited neon and, in addition, from N_2_ excitation. A similar effect is observed in the argon environment; however, the weak emission from excited *[Ru(bpy)_3_]^2+^ ion (620 nm) is still visible. The ion emission in the neon environment is not reported due to a strong Ne emission. The observed nitrogen emission can be caused by N_2_ molecules absorbed on the surface of the material [37].

**Figure 19:**
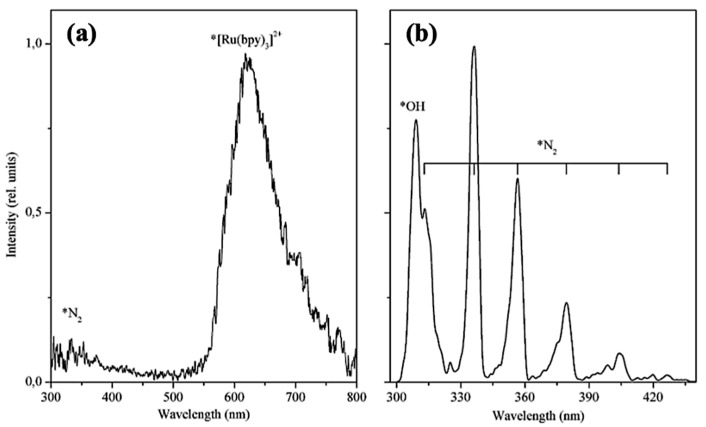
TL spectra of [Ru(bpy)_3_]Cl_2_: (**a**) in O_2_ atmosphere and (**b**) at 300–440 nm during injection of Ar under pressure up to 130 kPa. The emission peaks corresponding to the excited gases and ions are marked with *N_2_, *[Ru(bpy)_3_]^2+^, *OH symbols. Reprinted with permission from Elsevier [37].

The TL spectrum was also collected in O_2_ atmosphere (Figure 19). The reported emission from N_2_ was suppressed, while the emission from excited ion *[Ru(bpy)_3_]^2+^ was unchanged. On this basis, it was found that the excitation of the complex is not caused by absorption of the energy emitted by the excited N_2_ molecules.

### 5.4. TL and PL of the Manganese Complex

**Figure 20:**
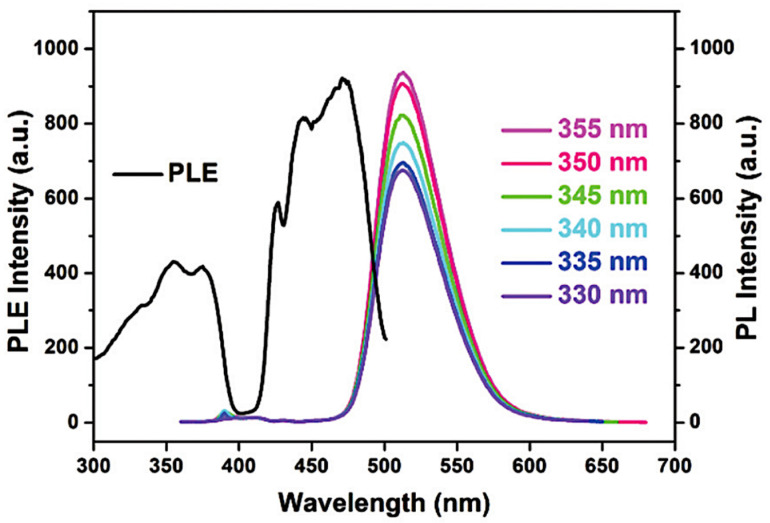
Excitation and photoluminescence spectra of the manganese(II) complex [34].

The excitation spectrum of the Mn(Ph_3_PO)Br_2_ contains a broad band between 250 and 350 nm. It is caused by π–π* transition in the compound [25]. Photoluminescence of the manganese(II) complex could be explained by using ligand field theory. The maximum emission is recorded at 510 nm, which is caused by ^4^T_1_ → ^6^A_1_ transition [9]. As a result of the mechanical interaction, green emission is observed. The excitation and PL spectra are presented below (Figure 20).

The photoluminescence efficiency of the manganese(II) complex can be affected by doping. HBr-doped compounds exhibit brighter emission and higher efficiency. On the other hand, a compound doped with HgCl_2_ performs weaker photoluminescence than an undoped complex. The decrease in emission could be caused by the presence of chlorine ions, which provide more non-radiative decay pathways [25].

The emission also decreases with the increase of surrounded gas pressure [40,41,42]. The measurements were performed with a modified Drickamer Type I high-pressure optical cell.

**Figure 21:**
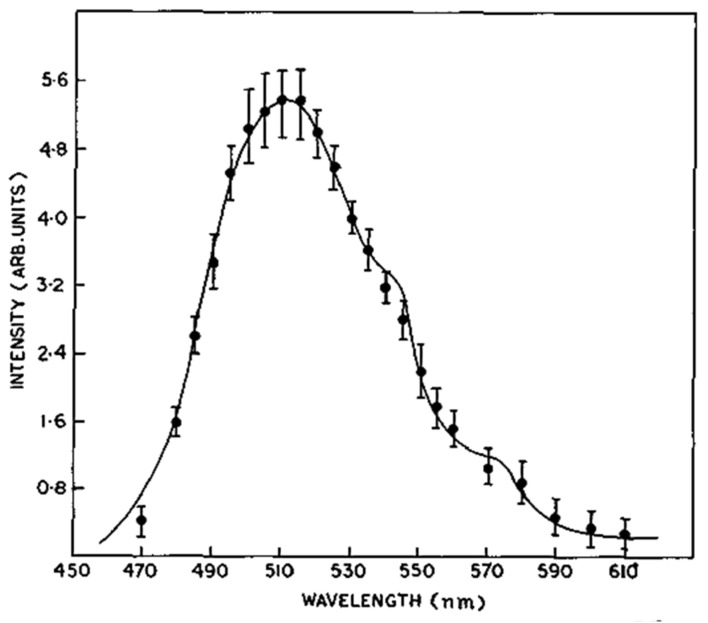
Photoluminescence (solid line) and triboluminescence (points) spectra of Mn(Ph_3_PO)_2_Br_2_. Reprinted with permission from Elsevier [41].

Mn(Ph_3_PO)_2_Br_2_ complex shows a strong triboluminescent. Emission maximum occurs at 510 nm. Triboluminescence spectrum shape is conforming to the photoluminescence spectrum (Figure 21) [41]. The emission lifetime of triboluminescence is equal to 602 μs [9]. Measurements were performed using a specially designed system described in Section 7.

**Figure 22:**
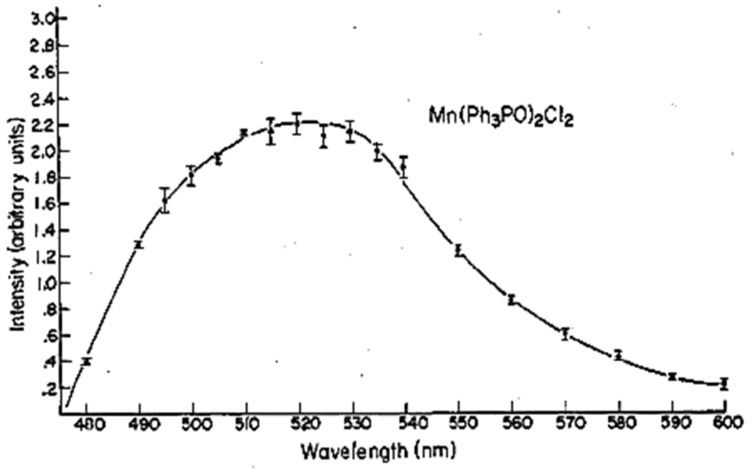
Triboluminescence spectra of Mn(Ph_3_PO)_2_Br_2_ [41].

Ligands in the complex structure have a significant impact on the luminescent properties. Interestingly, changing from Br^−^ ions to Cl^−^ causes the disappearance of the photoluminescence properties, although Mn(Ph_3_PO)_2_Cl_2_ still possesses efficient triboluminescent properties (Figure 22). For the manganese(II) complex with chlorine anions as a ligand emission maximum is shifted to 520 nm [9].

## 6. Various Stimuli Affecting the Triboluminescence

The common method for TL measurements utilizes a drop tower, where a falling object with defined weight is a cracking sample. Then, generated radiation is collected by a dedicated spectrometer. The details of the TL measurement system are described in Section 7.

**Figure 23:**
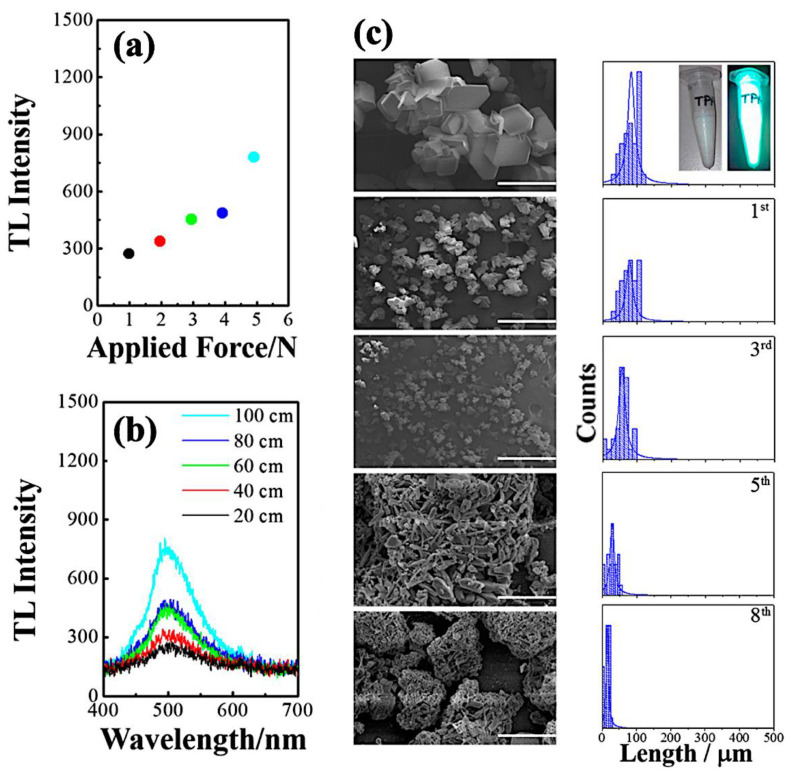
(**a**) TL intensity values vs. the applied drop height [28]; (**b**) TL profile changes due to the various h [28]; (**c**) SEM images (scale bar = 400 μm) and corresponding particle size distribution of the Cu(I) complex before and after mechanical action; top inset shows images of crystals before and after UV light exposure. Reprinted (adapted) with permission from (J. Phys. Chem. C 2017, 121, 21, 11709–11716). Copyright (2017) American Chemical Society [28].

Regardless of the impact force, the maximum intensities oscillate at wavelength values close to 496 nm. The spectrum shape of [Cu(NCS)(py)_2_(PPh_3_)] complex also remains unchanged. For the larger impact force, a higher intensity of the triboluminescence is observed. Between the smaller applied force (0.98 N) and the largest one (4.98 N), more than twice higher TL intensity was observed (Figure 23) [28,42].

Moreover, it has been shown that the TL emission depends on the crystallites size [28]. Each successive hit causes fragmentation of the crystalline material, which reflects the decrease of the triboluminescence intensity during the subsequent attempts [28]. The SEM pictures of the crystalline particles captured before the TL measurement and after the first, third, fifth, and eighth attempts are shown in Figure 23c. A Gaussian distribution was fitted for mean diameters, yielding results of 120 ± 20, 100 ± 15, 80 ± 12, 50 ± 10, and 30 ± 8 µm for the abovementioned hit numbers, respectively.

Combining the polymer matrix with photoluminescent and triboluminescent compounds, and their influence on the examination of optical properties has been recently reported in the literature [28,42]. It was extensively investigated what kind of effect may influence the triboluminescence phenomenon when placing the [Cu(NCS)(py)_2_(PPh_3_)] complex in another material. The Cu(I) complex was used in composites with different polymers and also placed inside the hydrogels structures [28,42].

### 6.1. Polymer Mats Coated with a Triboluminescent Compound

**Figure 24:**
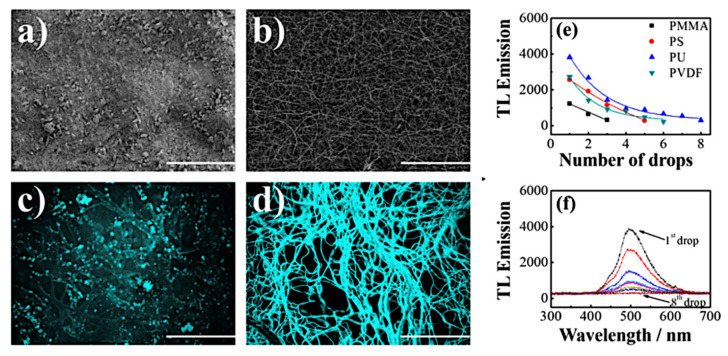
SEM (**a**,**b**) and FM (**c**,**d**) images (scale bar = 200 μm) of the composite mats prepared by both surface impregnation (**a**,**c**) and blending (**b**,**d**) [28]; (**e**) TL emission of the composites prepared by PMMA, PS, PVDF, and PU as a function of the number of drops; (**f**) TL response of the PU composite. Reprinted (adapted) with permission from (J. Phys. Chem. C 2017, 121, 21, 11709–11716). Copyright (2017) American Chemical Society [28].

To create a material with the triboluminescent properties, crystalline particles of the Cu(I) complex were deposited on the surface of electrospinning mats, made of four polymers: poly(methyl methacrylate) (PMMA), polystyrene (PS), polyurethane (PU), and poly(vinylidene fluoride) (PVDF). Spectroscopic measurements of TL were performed with the same tower system, as in the case of pure crystalline material (Figure 24).

**Figure 25:**
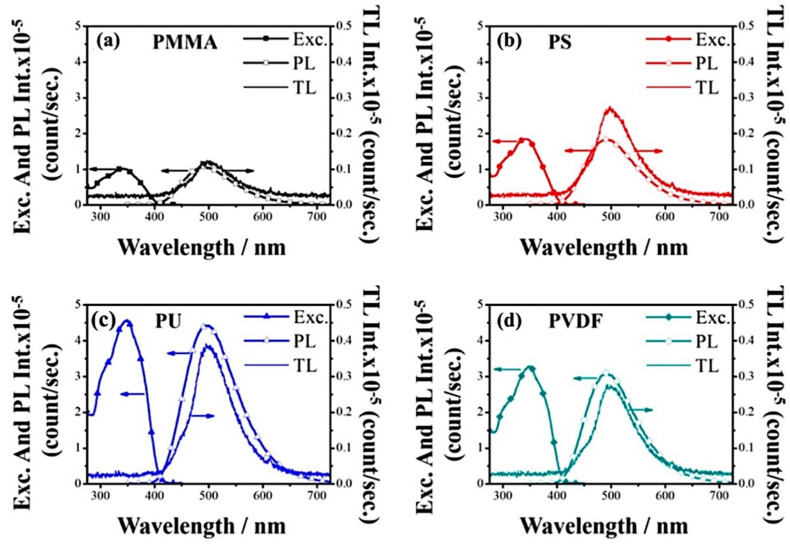
Excitation, PL, and TL emission spectra of the composite fiber mats: (**a**) PMMA, (**b**) PS, (**c**) PU, and (**d**) PVDF prepared by surface impregnation concerning the chemical structure of the polymer. Reprinted (adapted) with permission from (J. Phys. Chem. C 2017, 121, 21, 11709–11716). Copyright (2017) American Chemical Society [28].

Depending on the used polymer, different emission intensities were obtained. Triboluminescence intensities for the first impact are 1200, 2500, 4000, and 2750 counts per second for materials based on PMMA, PS, PU, and PVDF, respectively [28]. For all used composites (polymer mats/copper complex crystals), the triboluminescence signal is visible when the same sample is mechanically treated several times. For PMMA, PS, PVDF, and PU, triboluminescence response was observed for three, five, six, and eight impacts, respectively. For all of the subsequent samples, maxima and spectra shape are the same, but the intensity gradually decreases as the composite becomes quenched [28]. In addition, composite mats were also fabricated by another method. In this alternative approach, called blending, the [Cu(NCS)(py)_2_(PPh_3_)] crystals were dissolved in a polymer solution, and the homogeneous mixture was used to make electrospinning mats. Interestingly, the composites prepared by the blending method exhibited none of TL response [42]. Probably, during the dissolving process, the molecules of the copper complex dispersed in the polymer, which led to the loss of the long-range crystal structure. Differences between these two techniques are also visible in the SEM pictures (Figure 25a,b) and under a fluorescence microscope (Figure 25c,d). During imaging with fluorescence microscopy (FM, λ_exc._ = 365 nm), the samples of polymer substrates exhibited no luminescent properties, so the acquired colorful emission visible in the photos comes from the copper(I) complex. The surface impregnation process (Figure 25c) leads to creating local areas with higher intense emission, which is caused by advanced aggregation and complex particle deposition. For comparison, a composite obtained by blending (Figure 25d) shows a homogeneous luminescence over the entire surface of the fibers, which indicates dispersion of the copper(I) complex in the polymer matrix at the molecular level. This result implies an important connection between the crystalline structure and triboluminescent properties [28]. The PU fibers were the thinnest ones among the tested polymers. This probably contributed to the decrease in interfiber space, reduction of the sieving effect, and as a consequence, the deposition of more crystals. It is also possible that chemical affinity between the PU matrix and the copper(I) complex occurred [28]. The summarized parameters of enriched polymer triboluminescent mats are presented in Table 5.

In conclusion, it has been shown that the surface impregnation technique makes it possible to prepare homogeneous triboluminescent composites, where light emission depends on the polymer substrate type. Out of the used composites, the best triboluminescence properties were given by surface-impregnated PU. This shows that these materials can be applied in practice as, e.g., pressure sensors [11].

Apart from plane polymer mats, it is possible to receive three-dimensional polymeric structures with triboluminescence emission [43]. Hydrogels can be used for this purpose. Samples were synthesized using acrylamide (AA), and its derivatives as monomer.

Solutions of N-(hydroxymethyl)acrylamide (NHMA) and N-isopropylacrylamide (NIPAM) were combined with N,N′-methylenebisacrylamide (MBA) and ammonium persulphate (APS) as a reaction initiator, respectively. N,N,N′,N′-tetramethylethyldiamine (TEMED) was used as a cross-linker of polymers. Then, the Cu(I) complex crystals were added. The solutions were poured into a Petri dish and left to polymerize overnight at room temperature [42].

**Figure 26:**
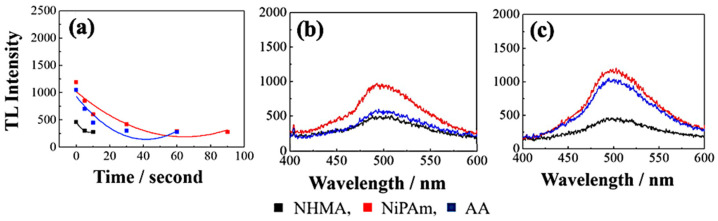
TL intensity vs. water treatment duration presentation (**a**). Emission spectra before (**b**) and after (**c**) water treatment of hydrogel samples [42].

The performed approach affected the TL properties. Namely, the growth of triboluminescence intensity was observed for all types of hydrogel samples compared to the pure Cu(I) complex. Moreover, the luminescence was more intense in the case of water treatment. The brighter light was emitted if the gel was placed underwater in a shorter time. Among monomers used in synthesis the NIPAM one was stood out, whose polymer matrix with Cu(I) complex achieved the highest TL emission time from all the investigated samples (Figure 26). NIPAM and ligands of the copper(I) complex have typical hydrophobic properties. Hence, such an approach may enhance the total emission time [42].

The triboluminescence aberrations can also come from the optical properties of the utilized polymer medium, which could easily propagate, scatter, or refract the emitted light. Hydrogels have greater optical density and viscosity compared to the surrounding air environment. These parameters’ growth intensifies triboluminescence emission. Moreover, the Cu(I) complex crystals suspended in the gel are separated from each other and kept a crystalline structure [42].

### 6.2. Dopant Implementation

Triboluminescent properties can be modified by the inclusion of organic dopants during the synthesis. Zeng X.R. et al. reported on a morphine-doped EuD_4_TEA complex exhibiting eight times higher triboluminescence than pure sample [43]. Moreover, the effect of piperine, DMMP, and TEPS doping was widely studied by Fontenot R.S. et al., who reported the influence of the dopants on TL yield, crystals morphology, synthesis time, and TL decay time [20]. The implementation of the piperine, which had been chosen due to its similarity to morphine, does not have a significant influence on the TL properties of the material. However, DMMP-doped material exhibits a 55% higher TL yield, whereas TEPS inclusion quenches the emission by 46% [20].

The measurements of photo- and triboluminescence show no influence of dopants on spectra. For both experiments shifts of the characteristic emission peaks are not observed. According to the authors, implemented dopants play the role of the co-activator or quenching agents [20].

Broad possibilities of the complex properties modification by doping with organic compounds significantly extend the implementation perspective. Even inclusion of unusual substances such as common multivitamin remarkably affects TL properties [23]. The influence of the series of organic compounds was widely described by Fontenot R.S. et al. [44].

## 7. Experimental Setup for the Triboluminescence Measurements

**Figure 27:**
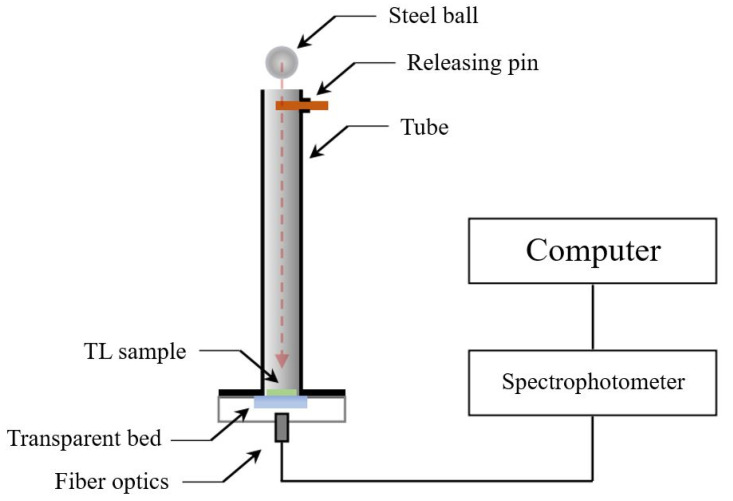
Representation of a drop tower used for the measurement of triboluminescence properties [36].

The TL measurement setup contains two fundamental elements: the first one is responsible for the force application and the second allows to perform the emitted radiation analysis. Although the TL measurement’s methodology is simple and the triboluminescent compounds are omnipresent, there are no commercially available setups for this type of experiment. It causes the need for a framework design and manufacturing by each of the research groups. The most commonly used type of triboluminescence measurement system is based on a drop tower in which the dropping ball constitutes a source of the mechanical force (Figure 27). The potential energy of the ball changes into kinetic energy, which is then transferred to the sample. By changing the height of the drop, it is possible to manipulate the energy of the interaction. The mechanical force implementation causes light emission. The emitted light is transferred via a fiber optic to a spectrometer, where induced radiation is collected and analyzed [36].

The undoubted advantage of the presented type of measurement system is an uncomplicated construction and its low manufacturing cost. The tunable height of the drop makes it possible to measure the influence of the conditions on the induced emission. As indicated in the previous part of the paper, the applied mechanical force is one of the stimuli which straightforwardly influences the triboluminescence properties [10,28]. Due to the simple setup construction, its modification is feasible. The drop tower can be used for both crystal powder and matrices doped with triboluminescent compounds. The implementation of the sealing system can allow for TL measurements in pure gas conditions. The significant influence of this stimulus has been observed previously [45]. However, the [Cu(NSC)(py)_2_(PPh_3_)] complex has not yet been tested in this way. The pure gas measurements may become an essential element of the triboluminescence mechanism description.

## 8. Perspectives of TL Implementation

Triboluminescent compounds have a wide range of applications. One of the most promising is designing a new group of mechanical force and stress sensors based on TL materials. An exemplary sensing system described by D. Olawale et al. is based on a manganese-doped zinc sulfide (ZnS:Mn) and can be used for concrete elements’ damage monitoring [11]. The proposed solution uses the connected fiber optics system, which is covered with the TL material in specific areas. The structural damages lead to light generation, which is, in the next step, detected by an electronic system monitoring the whole network of fiber optics. The proposed solution may have particular use in registering seismic damages of buildings [11].

Moreover, wind flow can be used to achieve a mechanoluminescence response. Jeong et al. designed elastic-ML materials, which consists of the multicolored ZnS:Cu microparticles embedded in PDMS fiber. This composite can be applied in wind-activated, energetically independent displays and lightning systems [46].

N. Terasaki et al. researched mechanoluminescent nanoparticles, which can be useful in bioimaging. Followed by the triboluminescence generation with ultrasonic waves, the compound can monitor the work of the digestive system [13]. Triboluminescence can also be used as a light source for a fluorescent probe. Ceramic TL materials that stay inside the body, or cells activated by ultrasounds emit light, influence organic dye molecules [47]. Furthermore, using ultrasound techniques these compounds found the application in light-supported therapies [13]. Additionally, the triboluminescent compounds have potential application in catalyzing photosensitive reactions. A method has been developed to synthesize stable SAOE-Zr-TiO_2_ by TL generation in situ. The obtained hybrid material is based on europium doped strontium aluminate (SAOE) with TiO_2_ nanoparticles [12].

The process of designing and manufacturing many types of solutions, especially sensors, is primarily based on the well-known materials’ properties changes under the influence of an external stimulus. The development of TL-based devices and techniques is significantly straightened due to the unknown nature of the triboluminescence phenomenon. Obtaining new triboluminescent materials may shed new light on the possible definition of the mechanism of stress-induced light emission.

## 9. Conclusions

In this contribution, we have described the variety of transition metal coordination complexes, which are excellent examples to study the triboluminescent mechanism. In the article, we paid attention to synthesis routes, various spectroscopic product analyses, as well as TL and PL measurements discussion. Moreover, the triboluminescent properties dependent on external factors, for example, embedded in the polymer mats, were presented. We found that the described compounds do not require a complicated synthesis apparatus and procedure. It can be received in a common laboratory with a simple working fume hood. Mainly, the synthesis is just a one- or two-step procedure. As discussed in this short review compounds have appealing spectroscopic properties, such as significant and visible TL and PL, which leads to many practical applications. This could help in further research on the TL mechanism, which would allow defining universal explanation or clear division for all cases of transformation mechanical energy into the light in different class materials.

Development in this field is crucial for further implementation. Currently, there are promising results; however, without additional knowledge about the nature of the considered phenomenon, it is impossible to fully exploit the triboluminescence potential.

## Figures and Tables

**Table 1:** Geometry of the [Cu(NCS)(py)_2_(PPh_3_)] molecule [10].

Bond Length (Å)		Bond Angle (°)	
Cu-X	2.013(2)	X-Cu-N(ar)	99.58(8)
Cu-N(ar)	2.091(2)	X-Cu-P	106.70(6)
	2.070(1)	P-Cu-N(ar)	115.34(6)
Cu-P	2.1974(5)	N(ar)-Cu-N(ar)	100.37(7)
S-C	1.638(2)	Cu-N-C	157.4(2)
C-N	1.168(3)	N-C-S	179.5(2)

**Table 2:** The geometry of the EuD_4_TEA molecule [29].

Bond Length (Å)		Bond Angle (°)	
Eu1-O1	2.365(16)	Eu1-O8	2.366(16)
Eu1-O3	2.369(15)	Eu1-O6	2.386(16)
Eu1-O2	2.385(17)	Eu1-O7	2.401(16)
Eu1-O4	2.402(13)	Eu1-O5	2.421(17)
O8-Eu1-O6	109.7(6)	O3-Eu1-O6	70.3(6)
O3-Eu1-O7	121.5(5)	O8-Eu1-O7	72.3(6)
O2-Eu1-O4	70.2(6)	O1-Eu1-O4	75.1(5)
O2-Eu1-O5	71.2(6)	O1-Eu1-O5	71.2(6)

**Table 3:** [Ru(bpy)_3_]Cl_2_∙6H_2_O crystal data [33].

Crystal Data		Data Collection	
Chemical formula	C_30_H_24_N_6_RuCl_2_∙6H_2_O	T_min_, T_max_	0.676, 1.000
M_r_	748.62	No. of measured, independent and observed [I > 2σ(I)] reflections	11 174, 1220, 994
a, c (Å)	13.1383 (12), 20.995 (3)	R_int_	0.084
V (Å3)	3138.6 (6)	θ values (^o^)	θ_max_ = 27.5, θ_min_ = 3.7
F(000)	1536	(sin θ/λ)max (Å^−1^)	0.650
Dx (Mg m^−3^)	1.584	Refinement	
µ (mm^−1^)	0.72	R[F^2^ > 2σ(F^2^)], wR(F^2^), S	0.061, 0.153, 1.09
Crystal size (mm)	0.21 × 0.16 × 0.12	No. of reflections	1220
		∆p_max_, ∆p_min_ (e Å^−3^)	0.98, −0.56

**Table 4:** Geometry of the Mn(Ph_3_PO)_2_Br_2_ molecule [9].

Bond Length (Å)		Bond Angle (°)	
		O–Mn–O	101.7(2)
Mn–O	2.036(5)	O–Mn–Br	109.8(2)
	2.027(5)	O–Mn–Br	114.2(2)
Mn–Br	2.467(1)	O–Mn–Br	103.7(2)
	2.475(1)	O–Mn–Br	111.6(2)
		Br–Mn–Br	114.95(5)

**Table 5:** Photophysical properties for the [Cu(NCS)(py)_2_(PPh_3_)] complex and its composites with polymeric mats (Φ_f_—absolute fluorescence quantum yield, E_opt_—optical band gap, FWHM—full width at half-maximum, $\lambda_{em}^{TL}$ —maximum TL emission wavelength, $\lambda_{em}^{PL}$ —maximum PL emission wavelength) [28].

[Cu(NCS)(py)_2_(PPh_3_)]		$\lambda_{em}^{PL}\;(\mathrm{nm})$	FWHM^PL^	$\lambda_{em}^{TL}(\mathrm{nm})$	FWHM^TL^	E_opt_ (eV)	Φ_f_ (%)
solid	bare	490	113	500	90	2.92	98.0
surface-impregnated	PMMA	490	113	497	87	3.06	30.8
	PS	490	107	496	85	3.05	34.7
	PU	497	116	498	86	3.05	87.7
	PVDF	490	112	496	85	3.00	52.9
blended	PMMA	512	130	NA	NA	3.44	0.28
	PS	519	127	NA	NA	3.53	0.50
	PU	525	140	NA	NA	3.57	1.75
	PVDF	519	143	NA	NA	3.55	0.86

## Data Availability

Not applicable.
